# The Examination of the Stomach Contents

**Published:** 1913-11-08

**Authors:** 


### THE CLINICAL LABORATORY.*

### II.—The Examination of the Stomach Contents.

It is only within the last few years that an examination of the stomach contents has become a part of the ordinary routine investigation of cases of alimentary disease. This has been due not to the absence of laboratories, which were hardly necessary for the simple tests which have been in use for years on the Continent—and these old tests are still the most valuable—but to the fact that the passage of the stomach tube has obtained, at least among the laity, the reputation of being a very painful performance. Whilst nothing can make it a pleasant procedure, much can be done to remove the discomfort and nausea which are apt to accompany it. One of the first essentials is to get the confidence of the patient, and one finds, as a matter of fact, that most patients are much relieved when they hear that the only procedure contemplated is the passage of a soft tube and not the old stomach pump, which must have been a very painful and uncomfortable procedure to have lingered so long in the public memory. The best form of apparatus is also the simplest —viz., an ordinary soft rubber stomach tube connected by means of a glass junction with a continuation rubber tube of the same calibre. Forms of suction apparatus should not be used unless it is desired to remove samples of gastric mucosa for microscopical examination. The only contraindications to the use of the simple tube are aortic aneurysm (or even a suspicion of such), dyspnœa due to vascular or other cause, advanced and hopeless cachexia, where it would be useless, and, finally, actual hæmatemesis. The last of these forms a contraindication for a period of fourteen days after the hæmorrhage. Menstruation and pregnancy during the early months are not contraindications, although they serve to invalidate the results, as variations in secretion seem to be almost physiological under these conditions.

The passage of the tube demands very little skill, but it is remarkable that very few practitioners appear to realise that for a successful issue it is not desirable that the patient should attempt to swallow the tube during the whole of its passage. Place the patient in the sitting position with the head well forward, pass the end of the tube, which has been dipped into hot water (glycerin and other lubricants are unnecessary and even nauseating to the patient), well into the back of the pharynx. Tell the patient to swallow when the end of the tube has reached the level of the cricoid, then to stop swallowing, to close the lips but not the teeth, and to *breathe* hard through the nose. The tube can then be pushed straight into the stomach. The upper end of the tube is placed in some suitable vessel and the patient told to compress his abdominal muscles. The stomach contents will then be expressed into the vessel. If these directions be followed without fuss and flurry, without lubricants, without cocainising the pharynx, and without any complicated suction apparatus, success is almost assured, even with a very nervous patient. It is important to note that no water must be poured into the tube to obtain syphon action as this would invalidate the subsequent examinations. Much ingenuity has been wasted in the elaboration of test meals, but, here again, the best are the simplest and the oldest. The *test breakfast* consists of about two ounces of white bread or toast and ten to fifteen ounces of tea or water. This must be taken on an empty stomach in the morning and the contents removed one hour later. The *test dinner* is now used chiefly as a test for the motor power of the stomach. It consists of a plate of beef soup followed by six to eight ounces of beef steak and six ounces of mashed potatoes. The stomach should be *washed out* seven hours later, when it should be quite empty. The presence of food in the stomach after this lapse of time is the best and most delicate test of motor insufficiency.

### Examination of the Stomach Contents.

There are so many methods of examination and so many elaborations which have crept into the text-books on this speciality that the best course to pursue in these articles will be to outline what should be the irreducible minimum. The following points should be noticed:—

(1) *Quantity.*—The simple method of expression outlined above does not empty the stomach, therefore no deductions should be drawn from a diminution of the quantity. When more than 150 c.c. are removed it may be assumed that there is an increase which is due to either pyloric obstruction or to hypersecretion, often to both factors together.

(2) *Appearance and Consistency.*—The normal chyme is very much like thick gruel in appearance, but should flow along the tube quite easily. An excess of secretion renders it more fluid, when it very easily separates into two layers—food *débris* below and secretion above. In gastritis with excessive mucus production the consistency is very much increased, and one can lift up masses of food mixed with mucus by means of a straight glass rod. When there is also achylia the presence of large lumps of undigested bread mixed with a little slimy fluid is very characteristic.

(3) *Abnormal Additions* are chiefly blood, bile, and mucus. Blood in small quantity has little significance, especially if it be red in colour, for it is easily produced by the abrasion of the stomach tube. Bile has also little significance, as it is also commonly present in small quantity. When constant and associated with other signs of stasis it is significant of a stricture of the duodenum below the ampulla of Vater. *Mucus* is of great importance. In addition to the small quantity of mucus secreted by the normal stomach there is always some mucus derived from the mouth and pharynx,

* The first article appeared on October 25.

in some cases also from the respiratory tract. This mucus is easily recognised, as it is glairy, and is formed into small balls which float on the surface and contain small bubbles of air. An excess of true gastric mucus is intimately mixed with the chyme, prevents filtration, and cannot be mistaken. This is characteristic of gastric catarrh, but it must always be remembered that gastritis is usually secondary to some other disease, such as cirrhosis of the liver or to carcinoma. In fact gastric catarrh is under certain circumstances—a short clinical history—almost diagnostic of cancer.

### Chemical Examination.

For this purpose the filtrate from a test breakfast should be employed. The essential parts of the examination are (1) demonstration of the presence or absence of free hydrochloric acid, and (2) the titration of the free hydrochloric acid and of the "total acidity."

The demonstration of free HCl is most satisfactory when *fresh* Günzburg's reagent is used in the manner prescribed in all the text-books of physiology and clinical methods. This reagent is, however, very expensive and does not keep well and may be replaced by Congo paper, which is almost as satisfactory. What does the presence or absence of free HCl imply? Its presence is a good general indication that the secretory powers of the stomach are good, whilst its absence shows that secretion is *much* impaired. This may be due to chronic gastritis, to atrophy of the gastric mucosa owing to chronic sepsis, cachexia, or blood diseases. Taken alone it is of no value in the diagnosis or exclusion of gastric cancer, although in conjunction with other data it may be useful.

A knowledge of the presence or absence of free acid is an indispensable guide to the rational drug and dietetic treatment of all forms of gastric and intestinal dyspepsia.

Titration of "free HCl" and of "total acid." It is simplest to combine these titrations as follows:—10 c.c. of the filtrate are placed in a small beaker and two drops of an alcoholic solution of dimethylamidoazobenzol added as indicator. In the presence of free acid the solution will show a very definite pink or red colour. Decinormal soda solution is now added from a burette until the red colour vanishes. The reading on the burette is now recorded (say, 2.7 c.c.). One or two drops of a solution of phenolphthalein indicator are now added, and the titration is then continued until a definite pink colour appears. This shows that all the acid has been neutralised. The reading on the burette records the acidity (say, 4.5 c.c.). The results are usually recorded in terms of the number of centimetres of decinormal soda solution which are necessary to neutralise the free HCl and "total acid" in 100 c.c. of stomach contents. Thus the record of the above would read "Free HCl=27; total acid=45." The normal values range from 20 to 40 for the free HCl and from 40 to 60 for the "total acid."

It is very difficult to give a short summary of the deductions which may be drawn from the results of the above procedures. They are quite indispensable for a correct dietetic treatment of any case of dyspepsia, but as regards diagnosis it must be at once admitted that they are valuable only when considered in conjunction with other clinical data. This, however, applies to all laboratory methods. Complete absence of free acid with the total acid below 12 is an indication of achylia, but there are a few cases in very nervous individuals where the gastric secretion appears to be subject to psychical inhibition. They can be recognised by the subsequent examination of the fæces after Schmidt's test diet (absence of connective tissues from the fæces) and the result confirmed by subsequent test breakfasts. In regard to the early diagnosis of cancer all that can be said is that they are apt to be misleading if confidence is placed on the dicta of the earlier writers. "Occult" blood in the stools combined with "anacidity" is, apart from a few cases of achylia with a "friable" mucosa, almost diagnostic of cancer. "Occult" blood persisting after a fortnight's treatment should lead to a diagnosis or at least a strong suspicion of cancer, even when there is a normal or excessive quantity of acid in the stomach contents. The diagnostic significance of hyperchlorhydria is not yet clear. Sir Berkeley Moynihan's dictum that all cases of hyperacidity are due to chronic ulcer or to some other organic disease, such as gallstones or chronic appendicitis, has now much to support it. It may be assumed without much risk of error that a total acidity of over 80 is due to some organic lesion, usually to ulcer of the stomach or duodenum.

There are many other points of interest in gastric analysis, amongst which may be mentioned the examination for lactic acid after a special test meal, of importance in the diagnosis of cancer—the examination for ferments and of the state of starch digestion (which give valuable data for dietetic treatment), the naked-eye and microscopical examination of the deposit from washings of the fasting stomach, and, finally, bacteriological examination. The tests outlined above in detail should form the basis of any routine diagnostic procedure, whilst the latter are of a more special nature and indicated in special cases.

